# Strain-Dependent Fermentation Enhances the Bioactivity of *Gracilariopsis lemaneiformis* Polysaccharide-Rich Fractions

**DOI:** 10.3390/foods15183272

**Published:** 2026-09-16

**Authors:** Jiaqi Huang, Xiaofeng Chen, Han Zhang, Shixin Huang, Ling Wang, Zhiwen Wu, Peng Wu, Xu Tang

**Affiliations:** 1 Third Institute of Oceanography, Ministry of Natural Resources, Xiamen 361005, China; huangjiaqi@tio.org.cn (J.H.); chenxiaofeng2022@tio.org.cn (X.C.); 15227753693@163.com (H.Z.); sxhuang@tio.org.cn (S.H.); wangling@tio.org.cn (L.W.); wuzhiwen@tio.org.cn (Z.W.); 2 Fujian Provincial Key Laboratory of Island Conservation and Development, Island Research Center, Ministry of Natural Resources, Xiamen 361005, China

**Keywords:** lactic acid bacteria fermentation, *Gracilariopsis lemaneiformis*, polysaccharide-rich fraction, *Caenorhabditis elegans*, healthspan

## Abstract

Lactic acid bacterial fermentation offers a mild strategy for tailoring seaweed-derived carbohydrate fractions and strengthening their functional properties. In this study, polysaccharide-rich fractions were recovered from unfermented *Gracilariopsis lemaneiformis* (GP) and from biomass fermented with two marine-derived lactic acid bacteria, *Lacticaseibacillus casei* DS31 (GP-D) and *Lactiplantibacillus plantarum* HJ-S2 (GP-H). Fermentation changed the recovery of total and sulfated carbohydrates and altered monosaccharide profiles. In wild-type N2 worms, GP, GP-D, and GP-H extended mean lifespan by 8.61%, 14.70%, and 21.21%, respectively, and improved heat and oxidative stress survival (median survival +44% and +20% for GP-H, respectively), locomotion, redox homeostasis (SOD activity +19.5%, MDA content −73.4% for GP-H), and proteostasis-related phenotypes. Promoter–reporter assays, DAF-16::GFP localization, and transcriptomic profiling identified distinct treatment-associated stress-response programs. Lifespan extension also persisted in a daf-16 loss-of-function background, supporting contributions from complementary longevity networks. Collectively, strain-dependent fermentation reshaped *G. lemaneiformis* polysaccharide-rich fractions and enhanced their in vivo bioactivity, with GP-H producing the broadest improvement across the measured lifespan and healthspan endpoints.

## 1. Introduction

Population aging has intensified the search for dietary strategies that preserve physiological function and stress resilience. Aging reflects interacting changes in redox balance, proteostasis, mitochondrial performance, and cellular maintenance rather than failure of a single pathway [1,2]. These systems-level characteristics make *Caenorhabditis elegans* especially useful for screening food-derived bioactives because lifespan, stress survival, movement, reproduction, protein aggregation, and conserved regulatory responses can be evaluated within one model [3,4,5]. The model has become particularly valuable for polysaccharide evaluation because it links organismal longevity with healthspan, stress resistance, reporter activity, and conserved signaling within a short experimental cycle [6]. Natural polysaccharides are attractive candidates in this context because their composition and higher-order structure can be tuned by food processing and linked to antioxidant, metabolic, and cytoprotective functions.

*Gracilariopsis lemaneiformis* (syn. *Gracilaria lemaneiformis*) is an economically important red alga and a rich source of agar-type and sulfated galactan polysaccharides [7,8]. These macromolecules are dominated by galactose-based residues, including 3,6-anhydrogalactose, while sulfate substitution, molecular-size distribution, monosaccharide composition, and extraction conditions influence their physicochemical behavior and biological activity [7,8,9,10]. *G. lemaneiformis* polysaccharides have shown antioxidant activity and have already been associated with lifespan extension, reduced polyglutamine aggregation, and DAF-16 nuclear localization in *C. elegans* [9,10,11]. Enzymatic depolymerization and controlled degradation of *G. lemaneiformis* polysaccharides can further increase antioxidant and cytoprotective activity, emphasizing the dependence of bioactivity on molecular size and structural features [12,13]. The previous finding that an unfermented fraction increased mean lifespan by more than 16% at a high dose provides direct evidence that this seaweed is a relevant substrate for healthy-aging-oriented functional food development [11].

The functional performance of native seaweed polysaccharides can nevertheless be constrained by dense cell-wall organization, limited extractability, and heterogeneous molecular architecture. Lactic acid bacterial fermentation offers a food-compatible means of changing this architecture through acidification, substrate-specific enzymatic reactions, and microbial metabolism [14,15,16]. Across plant-derived materials, fermentation can modify carbohydrate recovery, molecular size, monosaccharide ratios, sulfate-associated composition, and antioxidant or immunomodulatory activity [14,15,16]. Because these effects depend on both the substrate and the microbial strain, two lactic acid bacteria applied to the same algal biomass may generate chemically and functionally distinct fractions rather than equivalent products.

Recent in vivo studies reinforce the importance of linking polysaccharide chemistry to whole-organism function. Fermented coix seed polysaccharides improved antioxidant enzyme activity and lifespan in *C. elegans* more strongly than the corresponding water-extracted polysaccharides [17]. Probiotic-fermented ginseng and a polysaccharide–lactic acid bacterium complex likewise improved stress resistance and aging-related phenotypes [18,19]. Independent studies of *Polygonatum cyrtonema* and *Lycium barbarum* polysaccharides reported lifespan and healthspan benefits accompanied by overlapping insulin/IGF-1, DAF-16, SKN-1, HSF-1, stress-response, and metabolic signatures [20,21,22]. Collectively, these findings indicate that food polysaccharides can influence multiple conserved maintenance systems rather than a single antioxidant endpoint.

Microbial and fermented-food studies further demonstrate that strain identity can shape the longevity response. Fermented cereal-derived gerobiotic combinations and individual probiotic strains produced pronounced strain-specific effects on healthy longevity [23,24]. More recent work with *Lactobacillus fermentum* WC2020, *Lacticaseibacillus rhamnosus* IDCC 3201, *Lacticaseibacillus casei* HY2782, and fermented tempeh linked lifespan or healthspan improvements to distinct combinations of JNK/MAPK signaling, antioxidant defense, immune and metabolic regulation, and intestinal-barrier maintenance [25,26,27,28]. These studies show that fermentation can enhance bioactivity, but they do not resolve how two defined strains remodel the same seaweed substrate or whether the resulting fractions produce distinct chemical, physiological, and transcriptional profiles.

A direct strain-to-strain comparison is particularly relevant for *G. lemaneiformis*. Fermentation of another red seaweed, *Porphyra yezoensis*, with *Lactiplantibacillus plantarum* or *Lacticaseibacillus casei* produced strain-dependent changes in acidity, soluble constituents, volatile compounds, and in vitro antioxidant capacity [29]. However, comparable studies that connect strain-specific remodeling of *G. lemaneiformis* carbohydrate fractions with lifespan, healthspan, proteostasis, and host gene-expression responses remain limited. Addressing this gap can clarify whether fermentation merely increases bulk carbohydrate recovery or generates functionally distinct preparations with different biological response profiles.

Here, marine-derived *L. plantarum* HJ-S2 and *L. casei* DS31 were used to ferment *G. lemaneiformis* biomass. Polysaccharide-rich fractions recovered before fermentation (GP) and after fermentation (GP-H and GP-D, respectively) were compared for carbohydrate composition and structural characteristics. Their effects were then evaluated in *C. elegans* using lifespan, thermal and oxidative stress survival, locomotion, reproduction, antioxidant status, polyglutamine aggregation, stress-responsive reporters, DAF-16 localization, and transcriptomic profiling.

## 2. Materials and Methods

### 2.1. Materials

Dried *Gracilariopsis lemaneiformis* powder was purchased from Xi’an Ruiying Biotechnology Co., Ltd., Xi’an, China. Yeast extract, agar, beef extract, and peptone were obtained from Sangon Biotech (Shanghai, China). A Protein Assay Kit (BCA Protein Assay Kit (Cat. No. PC0022, Beijing Solarbio Science & Technology Co., Ltd., Beijing, China) and superoxide dismutase (SOD, Cat. No. BC0170, Beijing Solarbio Science & Technology Co., Ltd., Beijing, China) and malondialdehyde (MDA, Cat. No. BC0020, Beijing Solarbio Science & Technology Co., Ltd., Beijing, China) assay kits were purchased from Beijing Solarbio Science & Technology Co., Ltd. Unless otherwise stated, all reagents were of analytical grade.

Wild-type N2 and the transgenic or mutant *C. elegans* strains AM141 (rmIs133 [unc-54p::Q40::YFP]), TJ356 (zIs356 [daf-16p::daf-16a/b::GFP + rol-6(su1006)]), CF1553 (muIs84 [sod-3p::GFP + rol-6(su1006)]), CL2166 (dvIs19 [gst-4p::GFP::NLS] III), and CF1038 [daf-16(mu86)] were obtained from the Caenorhabditis Genetics Center. Worms were maintained at 20 °C on nematode growth medium (NGM) plates seeded with live *Escherichia coli* OP50.

The seaweed fermentation medium contained *G. lemaneiformis* powder (10 g), yeast extract (3 g), and purified water (1 L); the initial pH was adjusted to 7.0.

The main instruments and equipment used in this study included a BP-10 pH meter (Sartorius, Göttingen, Germany), a GR60DF vertical autoclave sterilizer (Xiamen Zhiwei Instrument Co., Ltd., Xiamen, China), an XS105 DU analytical balance (Mettler-Toledo Instruments, Shanghai, China), a biochemical incubator (Ningbo Laifu Technology Co., Ltd., Ningbo, China), an MQT-60R shaking incubator (Shanghai Minquan Instrument Co., Ltd., Shanghai, China), an SW-CJ-IFD laminar-flow clean bench (Suzhou Purification Co., Ltd., Suzhou, China), an Alliance e2695 high-performance liquid chromatograph (Waters, Milford, MA, USA), a 3K15 Sigma benchtop high-speed refrigerated centrifuge (Sigma, Darmstadt, Germany), a SpectraMax^®^ M5 multi-mode microplate reader (Molecular Devices, San Jose, CA, USA), the Nicolet iS50 Fourier-transform infrared spectroscopy instrument (Thermo Fisher Scientific, Waltham, MA, USA), an Axio imager fluorescence microscope (Zeiss, Oberkochen, Germany), and an Alpha 2-4 LD plus freeze dryer (CHRIST, Hagen, Germany).

The composition of MRS broth used for bacterial activation is shown in Table 1.

### 2.2. Preparation of Polysaccharide-Rich Fractions

*Lacticaseibacillus casei* DS31 was isolated from the intestine of large yellow croaker (*Larimichthys crocea*), whereas *Lactiplantibacillus plantarum* HJ-S2 was isolated from the intestine of Blainville’s beaked whale (*Mesoplodon densirostris*). Both strains were maintained in the laboratory culture collection [30,31].

Each strain was activated in de Man, Rogosa and Sharpe (MRS) broth at 36 °C for 24 h. Cells were collected by centrifugation (4000× *g*, 10 min, room temperature), washed twice with sterile phosphate-buffered saline, and resuspended to an OD600 of 1.0 before inoculation into the seaweed medium. Fermentation proceeded statically at 36 °C for 72 h. The fermentation temperature of 36 °C was selected based on the optimal growth temperature of both marine-derived strains, and the 72 h duration was determined from preliminary time-course monitoring [30,31]. Apparent OD600, pH, and reducing-sugar concentration were recorded every 12 h. The fermented broth and an identically processed non-inoculated control were autoclaved at 121 °C for 20 min before extraction.

The autoclaved broth or control was extracted at 95 °C for 2 h with continuous stirring and centrifuged (10,000× *g*, 15 min, 4 °C). Three volumes of ice-cold absolute ethanol were added to the supernatant, and the mixture was held at 4 °C overnight. The precipitate was collected, redissolved in purified water, and treated with 15% (*w*/*v*) trichloroacetic acid at a 1:1 volume ratio. After incubation on ice for 30 min and centrifugation, the supernatant was reprecipitated with three volumes of ethanol. The final precipitates were lyophilized and designated GP-D (DS31-fermented), GP-H (HJ-S2-fermented), and GP (non-fermented control). Because the preparations were not fractionated to homogeneity, they are referred to throughout as polysaccharide-rich fractions rather than purified polysaccharides.

### 2.3. Carbohydrate Quantification and Physicochemical Characterization

#### 2.3.1. Determination of Total Polysaccharide and Sulfated Polysaccharide Contents

Total carbohydrate content was determined by the phenol–sulfuric acid method using glucose for calibration [32]. A 0.04 mg/mL glucose stock solution was used to prepare the calibration series. After addition of 1 mL of 6% phenol and 5 mL of concentrated sulfuric acid, the mixtures were incubated at room temperature for 20 min and absorbance was measured at 490 nm.

Sulfate content was estimated using the BaCl_2_–gelatin turbidimetric method [33]. Briefly, 50 mg of each fraction was hydrolyzed with 25 mL of 1 mol/L HCl at 100 °C for 5 h. After cooling, 1 mL of hydrolysate was mixed with 3 mL of BaCl_2_–gelatin reagent, incubated for 10 min, and measured at 360 nm.

#### 2.3.2. Monosaccharide Composition Analysis of Polysaccharides

Monosaccharide composition was analyzed using mannose, glucuronic acid, rhamnose, glucose, galactose, arabinose, xylose, and fucose as standards. Samples were hydrolyzed with 4 mol/L trifluoroacetic acid at 110 °C for 4 h and derivatized with 1-phenyl-3-methyl-5-pyrazolone following Liu et al. [34], with modifications. Residual TFA was removed by repeated methanol evaporation under nitrogen. The derivatized products were extracted with chloroform, and the aqueous phase was filtered through a 0.22 μm membrane before HPLC analysis.

HPLC separation was performed on a Symmetry Shield RP C18 column (5 μm, 250 × 4.6 mm) using 0.1 mol/L phosphate buffer (pH 6.7) and acetonitrile (85:15, *v*/*v*) at 0.8 mL/min and 30 °C. The injection volume was 10 μL, and derivatives were detected at 245 nm.

The limits of detection (LODs) and quantification (LOQs) were determined for each monosaccharide standard based on the standard deviation of the blank response (σ) and the slope of the calibration curve (S), with LOD = 3.3σ/S and LOQ = 10σ/S. The LOD values ranged from 0.02 to 0.08 μg/mL and LOQ values from 0.06 to 0.25 μg/mL across the eight monosaccharides analyzed (mannose, glucuronic acid, rhamnose, glucose, galactose, arabinose, xylose, and fucose), confirming sufficient sensitivity for the composition ranges observed in the samples.

#### 2.3.3. FT-IR Spectroscopy

Fourier-transform infrared (FT-IR) spectroscopy was used to characterize polysaccharide samples [35]. Polysaccharide powder (approximately 1 mg) was thoroughly mixed with dry KBr (approximately 100 mg) at a sample-to-KBr ratio of 1:100 (*w*/*w*), ground to a fine mixture in an agate mortar, and pressed into translucent pellets under 10 tons of pressure for 1 min. Spectra were recorded over the range of 400–4000 cm^−1^ at a resolution of 2 cm^−1^ using a deuterated triglycine sulfate (DTGS) detector. Each spectrum was acquired with 32 cumulative scans at a data interval of 0.5 cm^−1^. A background spectrum of pure KBr was collected under identical conditions and subtracted automatically. All measurements were performed at room temperature (25 ± 1 °C).

### 2.4. Effects of the Polysaccharide-Rich Fractions in C. elegans

#### 2.4.1. Lifespan Assay

Synchronized L4-stage N2 or CF1038 [daf-16(mu86)] worms were transferred to NGM plates containing GP, GP-D, or GP-H or to untreated control plates and maintained at 20 °C. Approximately 25 worms were included per group in each of five independent biological replicates. Animals were transferred daily to freshly prepared plates, and survival was scored until all animals had died. An animal was scored as dead when it failed to respond to gentle touch with a platinum wire. Animals that crawled off the plate or were accidentally damaged were censored at the time of loss. Lifespan distributions were compared using Kaplan–Meier analysis and the log-rank test.

#### 2.4.2. Reproduction Assay

For reproduction assays, one synchronized worm was placed on each NGM plate and transferred to a fresh treatment-matched plate every 24 h. The original plates were incubated for a further 24 h before progeny were counted. Approximately 25 animals were analyzed per group in each of five independent biological replicates, and counting continued until egg laying ceased.

#### 2.4.3. Motility Assay

For motility assays, synchronized worms were maintained on treatment-matched plates and transferred daily. Body bends and head thrashes were counted for 20 s on days 5, 8, and 10. Approximately 25 randomly selected worms were evaluated per group at each time point in each of five independent biological replicates.

#### 2.4.4. Stress-Resistance Assays

For oxidative-stress assays, animals pretreated through day 6 were transferred to NGM plates containing 6 mmol/L tert-butyl hydroperoxide and maintained at 20 °C. Approximately 25 animals were included per group in each of five independent biological replicates. Survival was recorded every 2 h until all animals had died.

For thermotolerance assays, day-6 animals were transferred to treatment-matched NGM plates at 35 °C. Approximately 25 animals were included per group in each of five independent biological replicates. Survival was recorded every 2 h [36].

#### 2.4.5. Determination of Antioxidant Enzyme Activity in Worms

After synchronization, L4-stage N2 worms were maintained on treatment-matched NGM plates for 5 days and transferred daily. Approximately 500 worms were collected per tube and centrifuged at 3000 rpm for 1 min. The pellet was resuspended in 400 μL phosphate-buffered saline and homogenized with beads at 60 Hz for 5 min, followed by centrifugation at 12,000 rpm for 5 min. Protein concentration in the supernatant was determined using a BCA assay, and SOD activity and MDA content (TBARS assay) were measured with commercial kits and normalized to protein content according to the manufacturers’ instructions.

#### 2.4.6. Fluorescence Quantification of Polyglutamine (polyQ) Aggregates

Synchronized L1-stage AM141 worms, which express Q40::YFP in body-wall muscle, were cultured for 48 h on NGM plates containing GP, GP-D, GP-H, or a vehicle control. Young adults were immobilized with 4 mmol/L levamisole and imaged using a YFP-compatible fluorescence filter set. Aggregate number and fluorescence intensity per worm were quantified in ImageJ (version 1.54f, NIH, USA) using identical acquisition and analysis settings across groups. Approximately 25 animals were analyzed per group in each of five independent biological replicates.

#### 2.4.7. DAF-16::GFP Nuclear Localization Assay (TJ356)

Synchronized L1-stage TJ356 worms were cultured for 48 h on treatment-matched NGM plates. Animals were immobilized with 4 mmol/L levamisole and imaged by fluorescence microscopy. DAF-16::GFP localization was classified using prespecified cytoplasmic, intermediate, and nuclear categories, and the proportion of animals in each category was compared among groups. Approximately 25 animals were analyzed per group in each of five independent biological replicates.

#### 2.4.8. Fluorescence Quantification of SOD-3::GFP and GST-4::GFP (CF1553 and CL2166)

The transcriptional reporters CL2166 (gst-4p::GFP::NLS) and CF1553 (sod-3p::GFP) were used to assess gst-4 and sod-3 promoter activity, respectively. Synchronized L1 larvae were cultured for 48 h on treatment-matched plates, immobilized with 4 mmol/L levamisole, and imaged using identical microscope settings. Relative GFP fluorescence was quantified using a consistent region-of-interest procedure. Approximately 25 animals were analyzed per group in each of five independent biological replicates.

#### 2.4.9. Transcriptomic Analysis

Total RNA was extracted from treatment-matched *C. elegans* samples using TRIzol reagent, with three biological replicates analyzed for each of the control, GP, GP-D, and GP-H groups (12 libraries in total). RNA concentration and purity were assessed with a NanoDrop 2000 spectrophotometer (Thermo Fisher Scientific, Wilmington, DE, USA), and integrity was evaluated using agarose gel electrophoresis and an Agilent 5300 system. Poly(A)+ RNA was enriched with oligo(dT) magnetic beads, and libraries were sequenced on an Illumina platform. Reads were filtered with fastp v0.23.4. Because the supplied analysis was based on de novo transcript reconstruction, clean reads were assembled with Trinity v2.8.5; transcript abundance was estimated with RSEM v1.3.1 and expressed as transcripts per million (TPM). Unigenes were annotated against GO, KEGG, EggNOG, NR, Swiss-Prot, and Pfam resources.

TPM values from the 12 libraries were transformed as log_2_(TPM + 1). Principal-component analysis used all 18,940 assembled unigenes, and sample similarity was summarized by Pearson correlation. Stress-, proteostasis-, autophagy-, and longevity-associated genes were selected according to the phenotypic and reporter assays and visualized across the four treatment groups. Grouped expression values are presented as mean ± SD in the corresponding figure, together with the pairwise significance annotations generated in the original analysis.

### 2.5. Statistical Analysis

Data are presented as mean ± SD. Except for pooled biochemical assays and transcriptomic sequencing, animal-based phenotypic and reporter assays comprised five independent biological replicates, with approximately 25 animals per group in each replicate. Two-group comparisons used an unpaired two-tailed Student *t*-test, whereas comparisons involving three or more groups used one-way ANOVA followed by Tukey’s test. Treatment-by-time data were analyzed by two-way ANOVA with multiplicity-adjusted post hoc comparisons. Lifespan and stress–survival curves were analyzed by the log-rank (Mantel–Cox) test. Categorical DAF-16::GFP localization data were analyzed by a chi-square test or Fisher’s exact test when expected counts were small. A two-sided *p* value < 0.05 was considered statistically significant.

## 3. Results and Discussion

### 3.1. Fermentation Behavior and Composition of the Recovered Fractions

**Figure 1:**
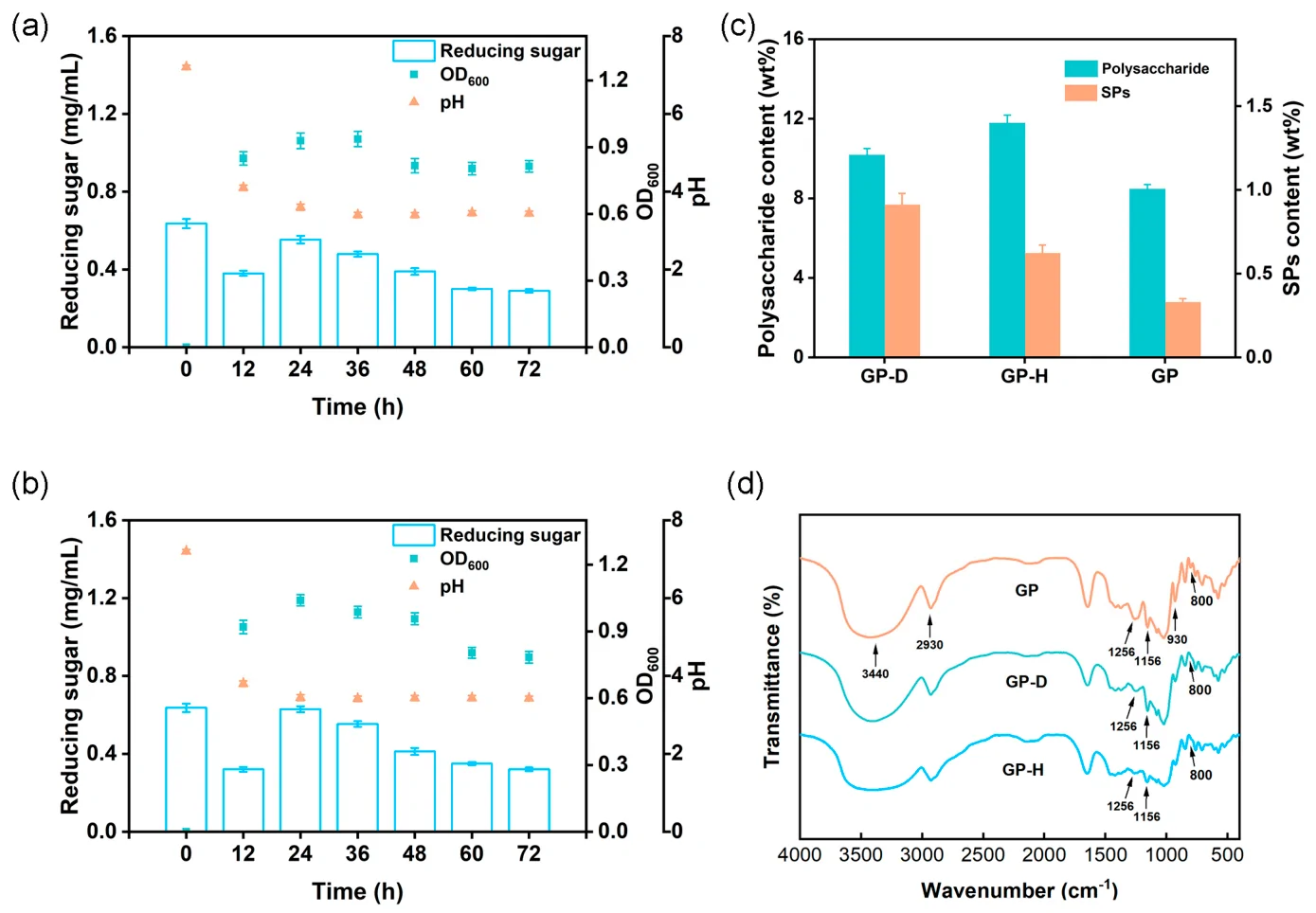
Fermentation behavior and composition of fractions recovered from *G. lemaneiformis*. Biomass-related OD_600_, pH, and reducing-sugar concentration during fermentation with (**a**) *L. casei* DS31 and (**b**) *L. plantarum* HJ−S2; (**c**) total carbohydrate and sulfate-associated contents of GP, GP−D, and GP−H; and (**d**) FT−IR spectra.

During the first 12 h, apparent OD_600_ increased while pH and reducing-sugar concentration decreased in both inoculated systems (Figure 1a,b). The coordinated changes were consistent with bacterial growth, acid production, and consumption of soluble carbohydrates. Between 12 and 24 h, the increase in apparent OD_600_ slowed while reducing-sugar concentration rose. This transient increase may reflect release of reducing ends or soluble sugars from the seaweed matrix at a rate that temporarily exceeded microbial uptake. After 24 h, apparent OD_600_ and pH approached plateaus, whereas reducing-sugar concentration declined gradually. The time course therefore supports active remodeling of the soluble carbohydrate pool rather than simple accumulation of bacterial biomass [15,16].

Fermentation also changed the amount of ethanol-precipitable carbohydrate-rich material. Total carbohydrate values increased from 8.45% in GP to 10.17% in GP-D and 11.78% in GP-H (Figure 1c), corresponding to relative increases of 20.4% and 39.4%, respectively. Sulfate-associated values increased from 0.33% in GP to 0.91% in GP-D and 0.62% in GP-H. Thus, the value for GP-D was 2.76-fold that of GP, while GP-H was 1.88-fold that of GP. The different rankings for total carbohydrate and sulfate-associated content indicate that the two strains did not simply increase recovery to the same extent. Instead, DS31 and HJ-S2 produced distinct compositional outcomes.

Microbial fermentation can modify carbohydrate recovery through acidification, carbohydrate-active enzymes, partial depolymerization, and microbial extracellular polymers [15,16]. These processes provide a coherent basis for the strain-dependent changes observed here. The FT-IR spectra retained the principal bands of hydroxyl-rich carbohydrates and sulfated galactans (Figure 1d) [7,9]. The broad and intense band near 3440 cm^−1^ is attributed to O–H stretching vibrations of hydroxyl groups and inter- and intramolecular hydrogen bonds [37]. The slightly reduced intensity in GP-D and GP-H relative to GP may reflect partial disruption of hydrogen-bond networks during fermentation-induced depolymerization, consistent with the observed changes in molecular accessibility. The band near 2930 cm^−1^ corresponds to C–H stretching vibrations of methyl and methylene groups in the carbohydrate backbone, and its relatively consistent position across samples indicates preservation of the basic carbon skeleton [37]. The band near 1256 cm^−1^ is assigned to S=O asymmetric stretching vibrations of sulfate ester groups, a characteristic feature of sulfated galactans [37,38]. The markedly higher intensity of this band in GP-D is consistent with its highest sulfate-associated content (0.91%), while GP-H shows an intermediate intensity matching its sulfate level (0.62%). The band near 930 cm^−1^ corresponds to C–O–C stretching of 3,6-anhydrogalactose, a diagnostic marker for agar-type red algal galactans [37,39]. Its preservation in all three samples confirms that the core agar structure was not destroyed by fermentation. The bands near 850 cm^−1^ and 800 cm^−1^ are associated with C–O–S bending vibrations of galactose-4-sulfate and pyranose ring skeletal vibrations, respectively [38,39]. The differential intensities of these bands among GP, GP-D, and GP-H further support strain-dependent remodeling of the sulfate substitution pattern and glycosidic linkage environment. Collectively, the shared band positions indicate preservation of the major galactan framework, while intensity differences—particularly in the sulfate-associated and fingerprint regions—are consistent with fermentation-induced changes in relative composition, degree of sulfation, and chemical environment [15,16].

### 3.2. Monosaccharide Composition

GP was dominated by glucose (53.93%) and galactose (32.47%), with rhamnose as the next most abundant monosaccharide (7.53%; Table 2). Fermentation with DS31 increased glucose by 10.42 percentage points and glucuronic acid by 0.87 percentage points, while galactose and rhamnose decreased by 6.32 and 4.62 percentage points. Fermentation with HJ-S2 increased glucose by 8.09 percentage points, glucuronic acid by 2.24 percentage points, and rhamnose by 1.23 percentage points, while galactose decreased by 9.19 percentage points. Mannose, xylose, and fucose also showed strain-specific nondetection patterns.

These shifts provide chemical evidence that the two fermentations remodeled different portions of the recovered carbohydrate pool. The higher relative abundance of glucuronic acid may increase anionic character and alter hydration or metal–ion interactions [10]. The larger sulfate-associated value in GP-D and the larger total carbohydrate value in GP-H further show that no single compositional descriptor explains the bioactivity ranking. Strain-specific acidification, enzyme repertoires, and extracellular-polysaccharide production offer complementary routes to the different profiles [15].

### 3.3. Effects of GP, GP-D, and GP-H on Lifespan and Stress Survival

**Figure 2:**
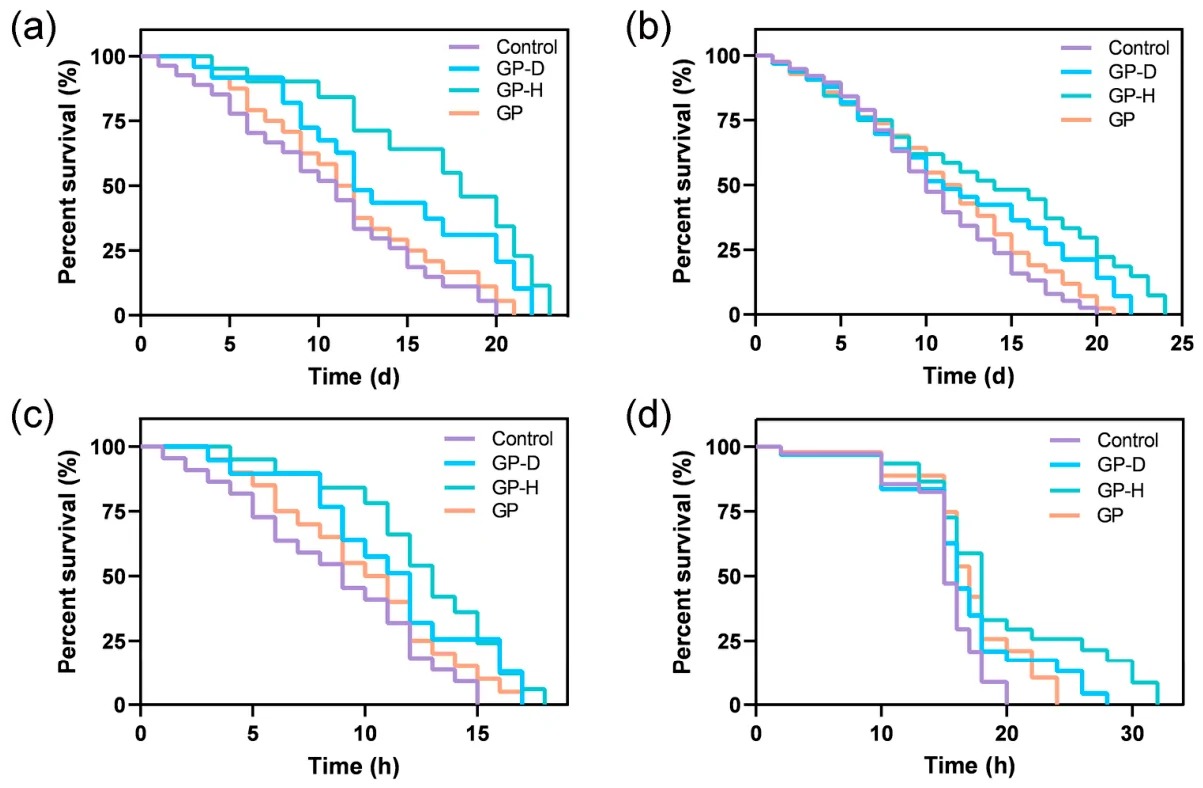
Effects of GP, GP-D, and GP-H on survival. Kaplan–Meier curves for (**a**) wild-type N2 and (**b**) CF1038 [*daf-16(mu86)*] animals; survival under (**c**) heat stress and (**d**) oxidative stress. Each assay comprised five independent biological replicates, with approximately 25 animals per group in each replicate.

Relative to the untreated control, mean lifespan increased by 8.61%, 14.70%, and 21.21% in the GP, GP-D, and GP-H groups, respectively (Figure 2a). The Kaplan–Meier analysis was based on five independent biological replicates, with approximately 25 animals per group in each replicate. Median survival times were 11, 12, 18, and 12 days, respectively. Pairwise log-rank tests versus control yielded *p* = 0.0256 for GP-D, *p* = 0.000434 for GP-H, and *p* = 0.4168 for GP. GP-H therefore produced the strongest survival phenotype, while GP-D generated a smaller but statistically significant shift.

The fermented fractions also improved survival during acute challenge (Figure 2c,d). Under heat stress, five independent biological replicates were analyzed, with approximately 25 animals per group in each replicate; median survival times were 9, 12, 13, and 10 h for the control, GP-D, GP-H, and GP groups, respectively. Log-rank *p* values versus control were 0.0411, 0.00279, and 0.2815 for GP-D, GP-H, and GP. Under oxidative stress, the same replicate design was used, and median survival times were 15, 16, 18, and 17 h for the control, GP-D, GP-H, and GP groups, respectively. Log-rank *p* values versus control were 0.0582, 0.000717, and 0.00291. GP-H was therefore the only fraction that significantly shifted both acute-stress curves and the wild-type lifespan curve under the tested conditions.

The magnitude and direction of the wild-type result are consistent with earlier work on *G. lemaneiformis* polysaccharide, which reported lifespan extension and reduced polyQ aggregation in *C. elegans* [11]. The present study extends that evidence by comparing non-fermented material with fractions recovered after two defined lactic acid bacterial fermentations. Related studies have reported enhanced longevity or antioxidant phenotypes after yeast fermentation of coix polysaccharides and probiotic fermentation of ginseng [17,18]. A 2025 Food Bioscience study likewise found that a Cistanche polysaccharide–probiotic complex improved antioxidant and aging-related phenotypes in worms [19]. Together, these findings position fermentation and polysaccharide–microbe combinations as promising food-processing strategies for healthy-aging-oriented ingredients.

In CF1038 [daf-16(mu86)] animals, mean lifespan increased by 10.07%, 15.44%, and 39.52% after GP, GP-D, and GP-H treatment, respectively (Figure 2b). Animals with median survival times of 10, 11, 14, and 11 days. Pairwise tests yielded *p* = 0.0407 for GP-D, *p* = 0.00384 for GP-H, and *p* = 0.2666 for GP. The significant GP-D and GP-H responses support a model in which DAF-16-linked regulation acts together with complementary longevity pathways. SKN-1 is one plausible parallel node because insulin-like signaling can regulate SKN-1 independently of DAF-16 [3,4].

### 3.4. Effects on Locomotion, Reproduction, and Antioxidant Status

**Figure 3:**
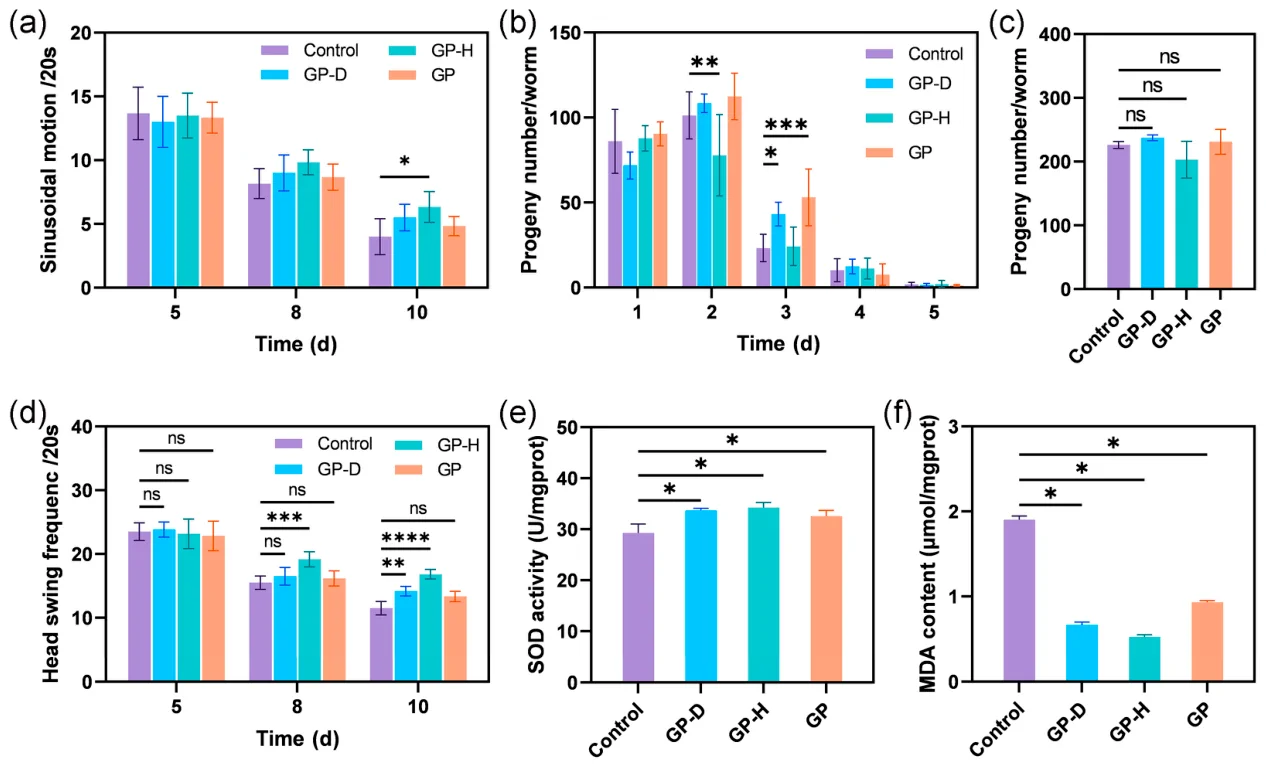
Effects of GP, GP-D, and GP-H on (**a**) body bends, (**b**) daily progeny production, (**c**) total brood size, (**d**) head thrashes, (**e**) SOD activity, and (**f**) MDA content in *C. elegans*. Reproduction and motility assays comprised five independent biological replicates, with approximately 25 animals per group in each replicate. The biochemical datasets contained four measurements per group for SOD and two measurements per group for MDA. * *p* < 0.05, ** *p* < 0.01, *** *p* < 0.001, and **** *p* < 0.0001 versus untreated control. ns, not significant.

The survival benefit was accompanied by selective improvements in locomotor performance. GP-H increased body-bend activity on day 10 and head-thrashing frequency on days 8 and 10, whereas GP-D increased head-thrashing frequency on day 10 (Figure 3a,d). These late-time-point effects are relevant because lifespan extension does not necessarily preserve physiological function throughout life [5]. The absence of uniform differences at earlier time points also argues against a nonspecific increase in movement across all ages.

Daily progeny production differed on days 2 and 3, but total brood size did not differ significantly among groups (Figure 3b,c). The lifespan and motility phenotypes therefore occurred without a detectable reduction in cumulative reproduction under the tested conditions, supporting improved late-life function without an evident reproduction–survival trade-off.

Biochemical measurements supported improved redox status. SOD activity increased by 8.41%, 19.52%, and 11.44% after GP, GP-H, and GP-D treatment, while MDA content decreased by 50.77%, 73.36%, and 63.73% (Figure 3e,f). Similar combinations of higher antioxidant-enzyme activity, lower lipid-peroxidation markers, and improved survival have been reported for Lycium and ginseng polysaccharides [40,41]. The biochemical and survival results therefore converge on enhanced redox resilience as an important component of the treatment response.

### 3.5. Effects on Proteostasis and Stress-Response Reporters

**Figure 4:**
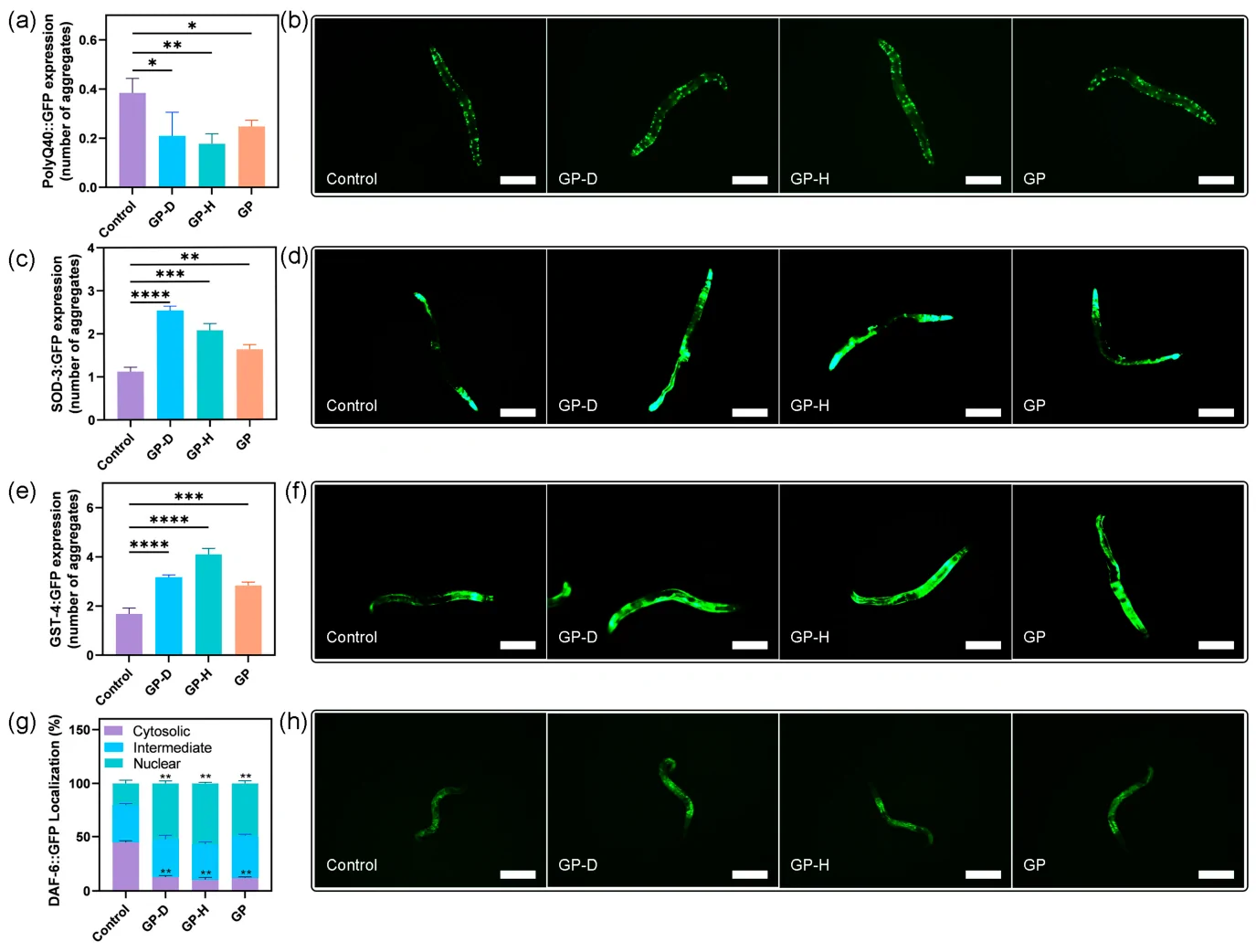
Effects of GP, GP-D, and GP-H on (**a**,**b**) Q40::YFP aggregate burden, (**c**,**d**) *sod-3* promoter–reporter fluorescence, (**e**,**f**) *gst-4* promoter–reporter fluorescence, and (**g**,**h**) DAF-16::GFP localization in *C. elegans*. Each assay comprised five independent biological replicates, with approximately 25 animals per group in each replicate. * *p* < 0.05, ** *p* < 0.01, *** *p* < 0.001, and **** *p* < 0.0001 versus untreated control. Asterisks indicate significant differences compared with the untreated control group.

GP, GP-D, and GP-H reduced Q40::YFP aggregate burden in AM141 animals (Figure 4a,b). This result reproduces the direction of the earlier *G. lemaneiformis* study and shows that fermentation retained or enhanced activity relevant to muscle proteostasis [11]. Together with the heat-survival and hsp-associated expression data, the reduction in aggregate burden supports improved protein-quality control in the treated animals.

The fractions also increased fluorescence driven by the sod-3 and gst-4 promoters (Figure 4c–f). These reporters capture transcriptional output associated with DAF-16- and SKN-1-linked stress programs, and their induction agrees with the increases in SOD activity and stress survival. GP, GP-D, and GP-H further increased the proportion of TJ356 animals with nuclear DAF-16::GFP (Figure 4g,h), confirming engagement of IIS/FOXO-associated regulation.

The reporter and mutant results together support a network interpretation. DAF-16 nuclear localization and sod-3 induction identify DAF-16-associated regulation as one component of the response. The significant lifespan extension in CF1038 animals further indicates that GP-D and GP-H recruit complementary cytoprotective programs. SKN-1 can act in parallel with DAF-16, and its roles in oxidative defense and longevity can be partly separable [3,4].

### 3.6. Transcriptomic Profiles

The 12 libraries corresponding to the four manuscript groups contained 36.08–42.01 million clean reads per sample. Q30 values ranged from 96.63% to 96.83%, and GC content ranged from 47.63% to 49.11%. De novo assembly yielded 23,717 transcripts and 18,940 unigenes, with unigene and transcript N50 values of 1805 and 1867 bp, respectively. BUSCO completeness reached 75.6% for the unigene set and 76.2% for the transcript set. A total of 16,845 unigenes (88.94%) received at least one database annotation, including 6827 unigenes (36.05%) assigned to KEGG. Together, these quality metrics provided a consistent basis for comparing treatment-associated expression profiles.

**Figure 5:**
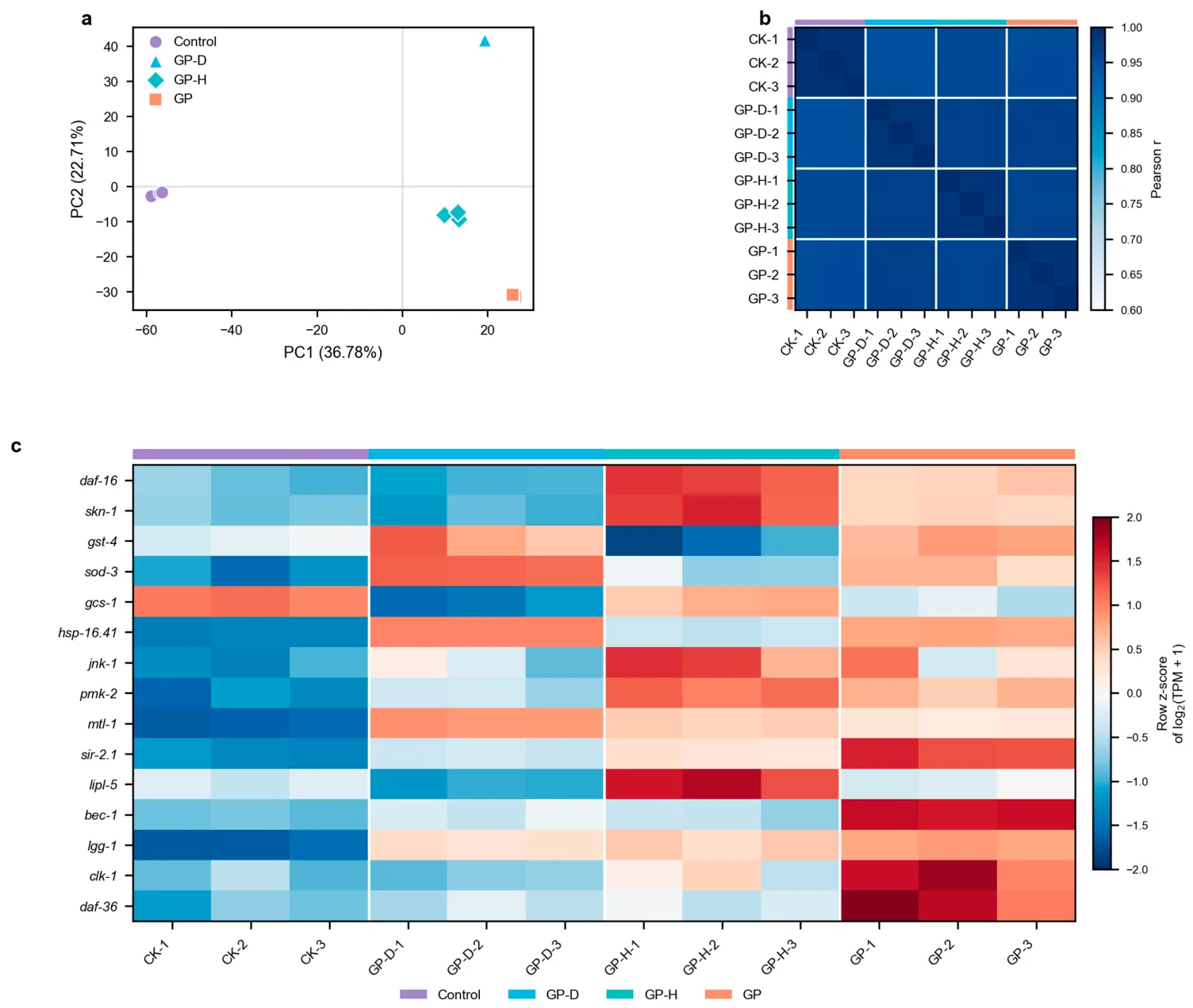
Transcriptomic profiles of *C. elegans* treated with GP, GP-D, or GP-H. (**a**) Principal-component analysis of log_2_(TPM + 1) values for all 18,940 assembled unigenes. (**b**) Pearson correlations among the same 12 libraries. Colored strips denote treatment groups. (**c**) Row-standardized log_2_(TPM + 1) values for selected stress- and longevity-associated genes. Each group contains three biological replicates.

Principal-component analysis of log_2_(TPM + 1) values separated the control, GP, GP-D, and GP-H samples, with PC1 and PC2 explaining 36.78% and 22.71% of the variance, respectively (Figure 5a). The three biological replicates clustered within each treatment, and within-group Pearson correlations ranged from 0.988 to 0.992 (Figure 5b). The clear group separation and high within-group correlations indicate reproducible treatment-associated transcriptomic structure.

The selected-gene heatmap further resolved distinct response profiles across the three fractions (Figure 5c). GP-H was characterized by higher abundance of daf-16, skn-1, jnk-1, and pmk-associated transcripts, whereas GP-D showed prominent increases in hsp-16-, sod-3-, and mtl-1-associated expression. GP produced a different profile enriched in sir-2.1-, autophagy-, and mitochondrial-function-associated transcripts. The treatments therefore generated complementary transcriptional programs rather than a uniform increase in one stress-response module.

### 3.7. Expression Patterns of Longevity- and Stress-Associated Genes

**Figure 6:**
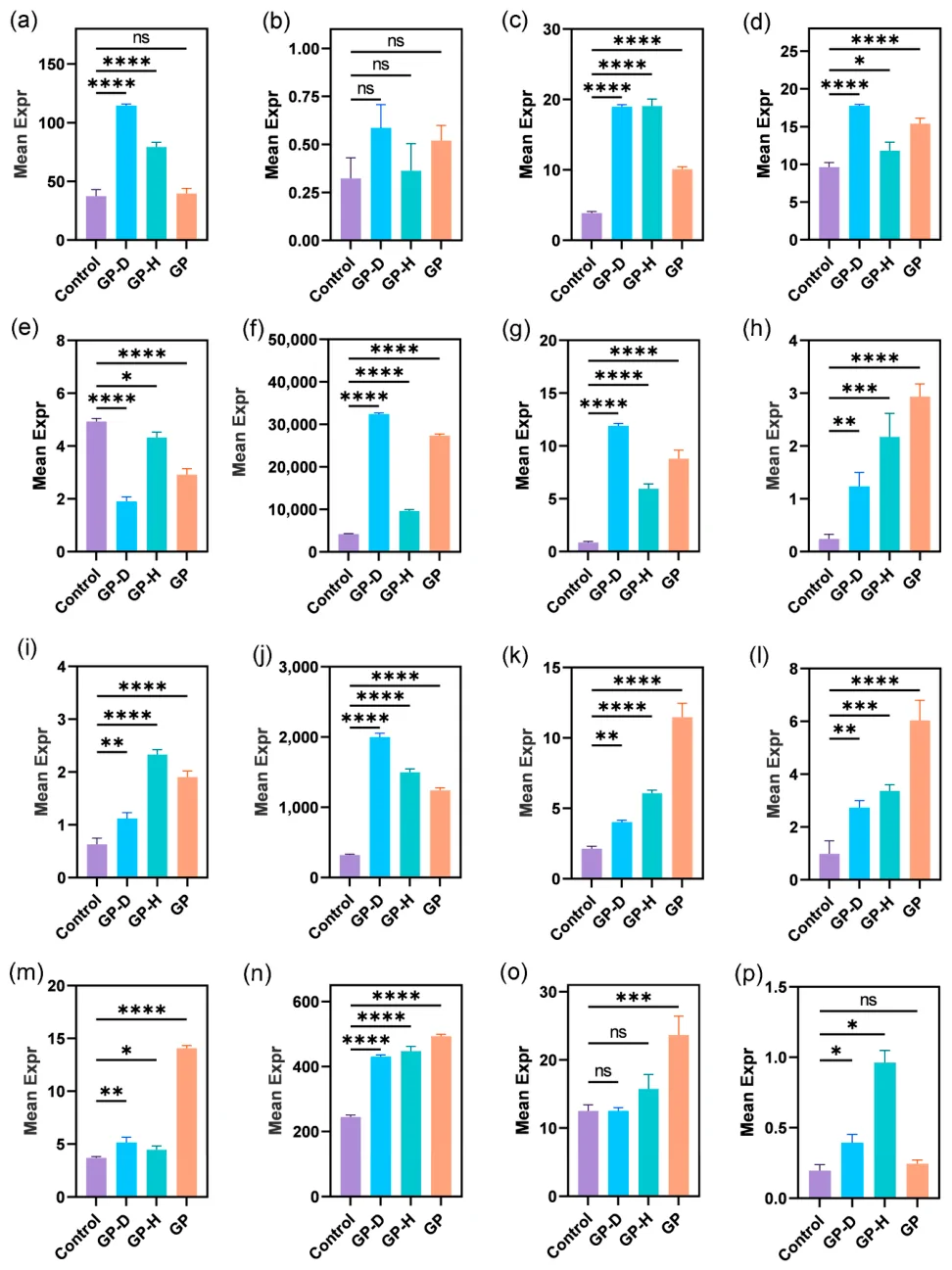
Expression of genes related to oxidative stress and aging: (**a**) daf-16, (**b**) skn-1, (**c**) gst-4, (**d**) sod-3, (**e**) gcs-1, (**f**) hsp-16.48, (**g**) hsp-16, (**h**) jnk-1, (**i**) pmk-1, (**j**) mtl-1, (**k**) sir-2.1, and (**l**) lipl-5; autophagy-related genes (**m**) bec-1 and (**n**) lgg-1; and mitochondrial-function-related genes (**o**) clk-1 and (**p**) mev-1. Bars show mean ± SD. Compared with the untreated control: * *p* < 0.05, ** *p* < 0.01, *** *p* < 0.001, and **** *p* < 0.0001; ns, not significant.

Expression profiling of 16 genes revealed clear treatment-specific responses across antioxidant, proteostasis, signaling, autophagy, and mitochondrial modules (Figure 6). GP-D and GP-H markedly increased daf-16 expression, whereas skn-1 remained comparatively stable. The three polysaccharide fractions also increased gst-4 and sod-3 expression, with the fermented fractions producing the strongest responses in these central antioxidant-defense genes.

The stress- and proteostasis-associated genes showed complementary rankings. GP-D produced the highest hsp-16.48- and hsp-16-associated expression, while GP-H and GP generated stronger responses in several JNK/p38-associated, metallothionein, and sirtuin-linked genes. These patterns parallel the improved heat and oxidative stress survival, increased SOD activity, reduced MDA content, and lower polyQ aggregate burden observed in the phenotypic assays.

Autophagy- and mitochondrial-function-associated genes were also responsive. bec-1 and lgg-1 increased across the treatment groups, with GP showing the largest bec-1-associated response. clk-1 was elevated by all three fractions, whereas mev-1 displayed a distinct fermentation-dependent pattern. The combined expression profile indicates that the treatments engaged multiple maintenance systems rather than relying on one isolated antioxidant target.

Overall, the expression data support a network-level response involving DAF-16-linked regulation, antioxidant defense, stress-activated signaling, proteostasis, autophagy, and mitochondrial remodeling. The distinct profiles of GP-D and GP-H are consistent with the strain-dependent chemical and physiological effects observed throughout the study.

### 3.8. Integrated Interpretation and Comparison with Related Studies

The central finding is that fermentation changed both the chemical profile and biological performance of the recovered *G. lemaneiformis* fractions in a strain-dependent manner. GP-D had the highest sulfate-associated value, whereas GP-H combined the highest total carbohydrate value with the broadest improvements in lifespan, stress resistance, locomotion, redox status, and proteostasis. This non-parallel ranking shows that functional performance cannot be predicted from one bulk-composition variable. A related study of *P. yezoensis* fermented with *L. plantarum* or *L. casei* likewise reported strain-dependent changes in acidity, soluble components, volatile profiles, and antioxidant capacity [29]. Studies using enzymatic or controlled degradation of *G. lemaneiformis* polysaccharides also found that structural remodeling and molecular-size reduction altered antioxidant performance [12,13]. Together, these results identify starter-strain selection and processing-induced structural change as decisive variables in the design of seaweed-derived functional ingredients.

The enhanced activity of the fermented fractions is also consistent with evidence from other carbohydrate-rich food matrices. Fermented coix seed polysaccharides had a lower molecular weight, stronger radical-scavenging activity, higher antioxidant enzyme activity, and greater lifespan benefit than water-extracted polysaccharides [17]. Probiotic-fermented ginseng improved oxidative-stress resistance and aging-related phenotypes in *C. elegans* [18], while a *Cistanche deserticola* polysaccharide–lactic acid bacterium complex alleviated multiple aging indicators [19]. *Polygonatum cyrtonema* polysaccharides similarly improved lifespan, motility, stress resistance, and conserved longevity-associated responses [20,21], and *Lycium barbarum* polysaccharides promoted lifespan and healthspan together with insulin/IGF-1-linked stress and lipid-metabolic remodeling [22]. These studies support a common food-bioprocessing principle: polysaccharide structure and processing history influence accessibility and the chemical environment of carbohydrate-rich materials, thereby shaping organism-level bioactivity.

The present redox and proteostasis data provide a coherent physiological explanation for the lifespan effect. GP-D and GP-H increased SOD activity, reduced MDA accumulation, improved survival during heat and oxidative challenge, and decreased Q40::YFP aggregate burden. The accompanying increases in sod-3 and gst-4 reporter signals connect these phenotypes to inducible cytoprotective responses. Unfermented *G. lemaneiformis* polysaccharides were previously reported to extend lifespan, reduce polyQ aggregation, and promote DAF-16 nuclear localization [11]. Fermentation therefore appears to build on an established activity of the algal substrate while producing stronger and more differentiated protective profiles.

DAF-16 emerged as an important regulatory node, but the mutant and expression data indicate a broader network. GP-D and GP-H promoted DAF-16 nuclear localization and increased daf-16-associated expression, yet lifespan extension persisted in daf-16(mu86) animals. This combination supports a model in which DAF-16 contributes to the wild-type response while parallel stress-regulatory systems maintain part of the benefit. Such network flexibility is consistent with the established interaction between DAF-16 and SKN-1 in oxidative-stress and longevity regulation [3,4]. It also resembles the strain-specific gerobiotic literature, in which bacterial or fermented-food treatments use overlapping but non-identical combinations of IIS, JNK/p38 MAPK, SKN-1, immune, and mitochondrial stress signaling [23,24,25,28].

The 16-gene panel further distinguishes the two fermented preparations. GP-D preferentially increased hsp-16-family expression and several redox-defense genes, matching its strong acute-stress and proteostasis phenotype. GP-H combined daf-16 induction with prominent changes in JNK/p38-associated, metallothionein, autophagy, and mitochondrial-function genes, consistent with its broader physiological performance. The increased bec-1 and lgg-1 expression provides a plausible link to enhanced cellular clearance, while changes in clk-1 and mev-1 connect the response to mitochondrial metabolism. The convergence of organismal, biochemical, reporter, and expression endpoints supports coordinated reinforcement of cellular maintenance rather than a single-gene effect.

Microbial carbohydrate products may also contribute to this integrated response. Bacterial colanic acid can alter mitochondrial dynamics and activate the mitochondrial unfolded-protein response in *C. elegans* [42]. Likewise, an exopolysaccharide isolated from heat-killed L. brevis improved longevity and proteostasis through responses involving protein quality control, autophagy, and lysosomal function [43]. Multi-omics analysis of L. rhamnosus IDCC 3201 connected host longevity with coordinated metabolic and immune regulation [26], whereas *L. casei* HY2782 improved the intestinal environment and barrier function together with lifespan [27]. Purified bacterial exopolysaccharides can also be sufficient to activate host detoxification and longevity programs involving FMO-2, NHR-49, and HLH-30 [44]. These precedents provide biological context for the strong hsp-, autophagy-, and mitochondrial-associated signatures observed here and suggest that fermentation-derived carbohydrate remodeling can engage conserved maintenance programs across distinct food and microbial systems.

A convergent cellular-maintenance response has also been observed with chemically defined dietary compounds. D-pinitol extended *C. elegans* lifespan while improving antioxidant defense, proteostasis, and HLH-30-dependent autophagy and mitophagy through interacting p38 MAPK–SKN-1, HSF-1, and clearance pathways [45]. The present parallel changes in SOD activity, MDA accumulation, Q40 aggregation, heat-shock-associated expression, autophagy-related genes, and mitochondrial-function genes follow a comparable systems-level pattern. This cross-study concordance supports the interpretation that the GP-H phenotype reflects coordinated reinforcement of cellular maintenance capacity rather than modulation of one isolated antioxidant target.

From a functional-food perspective, the study connects three levels of evidence within one experimental design: strain-specific fermentation chemistry, whole-organism lifespan and healthspan outcomes, and coordinated molecular responses. GP-H produced the most comprehensive benefit, whereas GP-D generated a distinct redox- and proteostasis-oriented profile. Together with recent studies showing that different probiotic strains and fermented foods engage distinct host-maintenance programs [25,26,27,28], this differentiation supports treating starter-strain identity as a controllable design variable rather than a generic processing input. Bioactivity-guided fractionation and structure–activity analysis can now build on these results to identify the carbohydrate features that best predict the desired functional profile.

The distinct outcomes produced by *L. plantarum* HJ-S2 and *L. casei* DS31 also warrant mechanistic consideration. *L. plantarum* species generally exhibits a versatile carbohydrate-utilization capacity, supported by a broad repertoire of carbohydrate-active enzymes, including glycoside hydrolases and phosphotransferase systems, and can rapidly acidify plant-derived substrates [46,47]. By contrast, *L. casei* displays distinct sugar-utilization patterns, substantial proteolytic activity, and different acidification characteristics [47,48]. These metabolic differences provide a plausible mechanistic basis for the observed strain-dependent changes observed in the present study. The broader carbohydrate-processing capacity of HJ-S2 may have promoted more extensive remodeling of the seaweed-derived carbohydrate substrate, thereby contributing to the higher total carbohydrate value observed in GP-H. In contrast, the distinct proteolytic and acidification profile of DS31 may have facilitated the release or enrichment of sulfate-associated components, consistent with the higher sulfate-associated value observed in GP-D. Moreover, because both HJ-S2 and DS31 were originally isolated from marine animal intestines [30,31], their marine origin may contribute to their adaptation to complex marine carbohydrate environments and, consequently, to their ability to remodel *G. lemaneiformis*-derived carbohydrate substrates.

## 4. Conclusions

This study shows that lactic acid bacterial fermentation can reshape *G. lemaneiformis* polysaccharide-rich fractions and enhance their in vivo functional activity. GP-D and GP-H generated distinct chemical and transcriptional profiles, and GP-H produced the broadest improvements in lifespan, stress resistance, locomotion, redox homeostasis, and proteostasis in *C. elegans*. Reporter localization, mutant survival, and selected-gene expression collectively support the involvement of interacting DAF-16-linked, stress-signaling, autophagy, and mitochondrial maintenance programs. Comparison with recent polysaccharide, probiotic, and dietary-compound studies further places the GP-H phenotype within a convergent biological model in which redox defense, proteostasis, autophagy, and mitochondrial regulation jointly sustain healthspan. These findings establish starter-strain selection as a practical route for tuning the biological performance of seaweed-derived carbohydrate ingredients and provide both a process-level principle and an organismal benchmark for their development in healthy-aging-oriented functional foods.

## Figures and Tables

**Table 1:** Composition of MRS broth per liter.

Component	Amount	Component	Amount
Peptone	10 g	Yeast extract	5 g
Beef extract	10 g	Diammonium hydrogen citrate	2 g
Glucose	20 g	Tween 80	1 mL
Sodium acetate	5 g	Dipotassium hydrogen phosphate (K_2_HPO_4_)	2 g
Magnesium sulfate (MgSO_4_)	0.58 g	Manganese sulfate (MnSO_4_)	0.25 g

**Table 2:** Monosaccharide composition of GP, GP-D, and GP-H.

Monosaccharide	GP (mol%)	GP-D (mol%)	GP-H (mol%)
Mannose	0.61 ± 0.04 ^b^	N.D.	1.14 ± 0.08 ^a^
Glucuronic acid	1.91 ± 0.12 ^c^	2.78 ± 0.15 ^b^	4.15 ± 0.21 ^a^
Rhamnose	7.53 ± 0.38 ^b^	2.91 ± 0.17 ^c^	8.76 ± 0.44 ^a^
Glucose	53.93 ± 1.62 ^b^	64.35 ± 1.93 ^a^	62.02 ± 1.86 ^a^
Galactose	32.47 ± 1.30 ^a^	26.15 ± 1.05 ^b^	23.28 ± 0.93 ^c^
Arabinose	1.31 ± 0.08 ^b^	2.01 ± 0.10 ^a^	0.65 ± 0.04 ^c^
Xylose	0.55 ± 0.03 ^b^	1.81 ± 0.09 ^a^	N.D.
Fucose	1.69 ± 0.08 ^a^	N.D.	N.D.

Data are presented as mean ± SD (*n* = 3). Different superscript letters within the same row indicate significant differences among groups by one-way ANOVA with Tukey’s post hoc test (*p* < 0.05). N.D., not detected under the analytical conditions used.

## Data Availability

The RNA-seq data generated in this study are not publicly available owing to intellectual property restrictions, but are available from the corresponding authors upon reasonable request. All other data supporting the findings are included in the article or available from the corresponding authors.

## References

[1] Dilberger B., Baumanns S., Schmitt F., Schmiedl T., Hardt M., Wenzel U., Eckert G.P. (2019). Mitochondrial oxidative stress impairs energy metabolism and reduces stress resistance and longevity of *C. elegans*. Oxidative Med. Cell. Longev..

[2] Zhu F.D., Chen X., Yu L., Hu M.L., Pan Y.R., Qin D.L., Wu J.M., Li L., Law B.Y.K., Wong V.K.W. (2023). Targeting autophagy to discover the Piper wallichii petroleum ether fraction exhibiting antiaging and anti-Alzheimer’s disease effects in *Caenorhabditis elegans*. Phytomedicine.

[3] Kirchweger B., Zwirchmayr J., Grienke U., Rollinger J.M. (2023). The role of *Caenorhabditis elegans* in the discovery of natural products for healthy aging. Nat. Prod. Rep..

[4] Mudd N., Liceaga A.M. (2022). *Caenorhabditis elegans* as an in vivo model for food bioactives: A review. Curr. Res. Food Sci..

[5] Sayed S.M.A., Siems K., Schmitz-Linneweber C., Luyten W., Saul N. (2021). Enhanced healthspan in *Caenorhabditis elegans* treated with extracts from the Traditional Chinese Medicine plants *Cuscuta chinensis* Lam. and *Eucommia ulmoides* Oliv. Front. Pharmacol..

[6] Zheng H.-M., Su C., Wang Z., Zhao L.-C., Li W. (2025). Review on potential of *Caenorhabditis elegans* as an anti-aging evaluation model for polysaccharides. Int. J. Biol. Macromol..

[7] Long X., Hu X., Liu S., Pan C., Chen S., Li L., Qi B., Yang X. (2021). Insights on preparation, structure and activities of *Gracilaria lemaneiformis* polysaccharide. Food Chem. X.

[8] Wang R., Wang J., Wang Z., Pan J., Sun S., Ma C., Tang X., Lv S., Zhai S., Zhao G. (2025). Extraction methods, structural characteristics, biological activities, and applications of the polysaccharides from *Gracilaria lemaneiformis*: A review. Int. J. Biol. Macromol..

[9] Wu S., Lu M., Wang S. (2017). Amylase-assisted extraction and antioxidant activity of polysaccharides from *Gracilaria lemaneiformis*. 3 Biotech.

[10] Fernandes P.A.R., Coimbra M.A. (2023). The antioxidant activity of polysaccharides: A structure–function relationship overview. Carbohydr. Polym..

[11] Wang X., Zhang Z., Zhou H., Sun X., Chen X., Xu N. (2019). The anti-aging effects of *Gracilaria lemaneiformis* polysaccharide in *Caenorhabditis elegans*. Int. J. Biol. Macromol..

[12] Fang T., Zhang X., Hu S., Yu Y., Sun X., Xu N. (2021). Enzymatic degradation of *Gracilariopsis lemaneiformis* polysaccharide and the antioxidant activity of its degradation products. Mar. Drugs.

[13] Long X., Hu X., Pan C., Xiang H., Chen S., Qi B., Liu S., Yang X. (2022). Antioxidant activity of *Gracilaria lemaneiformis* polysaccharide degradation based on Nrf-2/Keap-1 signaling pathway in HepG2 cells with oxidative stress induced by H_2_O_2_. Mar. Drugs.

[14] Hu T.G., Zhu W.L., Yu Y.S., Zou B., Xu Y.J., Xiao G.S., Wu J.J. (2022). The variation on structure and immunomodulatory activity of polysaccharide during the longan pulp fermentation. Int. J. Biol. Macromol..

[15] Liu S., Hu J., Zhong Y., Hu X., Yin J., Xiong T., Nie S., Xie M. (2024). A review: Effects of microbial fermentation on the structure and bioactivity of polysaccharides in plant-based foods. Food Chem..

[16] Wang Z., Zheng Y., Dai Y., Yang R., Zhao R., Sun G., Zhou W.W., Feng S., Feng Y., Li N. (2024). Effect of probiotic fermentation on the extraction rate and bioactivity of plant-based polysaccharides: A review. Innov. Food Sci. Emerg. Technol..

[17] Zhao D., Yan M., Xu H., Liang H., Zhang J., Li M., Wang C. (2023). Antioxidant and antiaging activity of fermented coix seed polysaccharides on *Caenorhabditis elegans*. Nutrients.

[18] Xu H.Y., Li Q.C., Zhou W.J., Zhang H.B., Chen Z.X., Peng N., Gong S.Y., Liu B., Zeng F. (2023). Anti-oxidative and anti-aging effects of probiotic fermented ginseng by modulating gut microbiota and metabolites in *Caenorhabditis elegans*. Plant Foods Hum. Nutr..

[19] Fang Z., Liu N., Fan Z., Wang W., Luo J., Qiu Y., Wang J., Li B. (2025). Cistanche deserticola polysaccharides–Companilactobacillus crustorum LBK 1001 complex relieves aging in *Caenorhabditis elegans*. Food Biosci..

[20] Zhang X., Chen Q., Chen L., Chen X., Ma Z. (2024). Anti-aging in *Caenorhabditis elegans* of polysaccharides from *Polygonatum cyrtonema* Hua. Molecules.

[21] Wang W., Yang Y., Hou T.-T., Yi S.-Y., Chen Y.-J., Li G.-S., Chen C.-W., Han B.-X., Ke J.-P., Liu D. (2023). *Polygonatum cyrtonema* Hua polysaccharides with antiaging and stress resistance efficacies in *Caenorhabditis elegans*. J. Food Biochem..

[22] Chen L., Yang Z., Chen X., Chen Q., Hao J., Ma Z. (2026). *Lycium barbarum* polysaccharides promote longevity and healthspan in *Caenorhabditis elegans* via insulin/IGF-1 signalling and lipid metabolic remodelling. Biogerontology.

[23] Govindhan T., Amirthalingam M., Duraisamy K., Cho J.H., Tawata S., Palanisamy S. (2023). Fermented cereal-origin gerobiotic cocktails promote healthy longevity in *Caenorhabditis elegans*. Food Funct..

[24] Zhang J., Zhao Y., Sun Z., Sun T. (2022). *Lacticaseibacillus rhamnosus* Probio-M9 extends the lifespan of *Caenorhabditis elegans*. Commun. Biol..

[25] Yang X., Chen J., Liao Z., Xia Z., Huang H., Huang J., Chen L., Fang X., Gao C., Wang J. (2024). *Lactobacillus fermentum* WC2020 increased the longevity of *Caenorhabditis elegans* via JNK-mediated antioxidant pathway. J. Food Sci..

[26] Lee D.J., Eor J.Y., Kwak M.J., Lee J., Kang A.N., Mun D., Choi H., Song M., Kim J.N., Kim J.M. (2024). Metabolic regulation of longevity and immune response in *Caenorhabditis elegans* by ingestion of *Lacticaseibacillus rhamnosus* IDCC 3201 using multi-omics analysis. J. Microbiol. Biotechnol..

[27] Bae W.-Y., Nguyen U.T.T., Le T.A.N., Tran S.H., Lee S., Hong S.-C., Cha K.H., Choi I.-D., Kang K. (2025). *Lacticaseibacillus casei* HY2782 improves the intestinal barrier and tract environment and ultimately prolongs the lifespan of *Caenorhabditis elegans*. Food Funct..

[28] Cho J., Cho C.-H., Joo Y.H., Wei C.-I., Lee S.-H. (2025). Tempeh, a fermented soybean, extends lifespan via MAPK pathway and improves healthy aging in *Caenorhabditis elegans*. Curr. Res. Food Sci..

[29] Gao T., Chen J., Xu J., Gu H., Zhao P., Wang W., Pan S., Tao Y., Wang H., Yang J. (2022). Screening of a Novel *Lactiplantibacillus plantarum* MMB-05 and *Lacticaseibacillus casei* Fermented Sandwich Seaweed Scraps: Chemical Composition, In Vitro Antioxidant, and Volatile Compounds Analysis by GC-IMS. Foods.

[30] Wan J., Wu P., Huang J., Huang S., Huang Q., Tang X. (2023). Characterization and evaluation of the cholesterol-lowering ability of *Lactiplantibacillus plantarum* HJ-S2 isolated from the intestine of Mesoplodon densirostris. World J. Microbiol. Biotechnol..

[31] Liu Y., Wu P., Li Y., Chen X., Wang L., Su Y., Luo L., Cai Y., Huang Q., Tang X. (2025). Probiotics and amelioration of periodontitis: Significant roles of *Lacticaseibacillus casei* DS31. Front. Microbiol..

[32] Zhang W.H., Wu J., Weng L., Zhang H., Zhang J., Wu A. (2020). An improved phenol–sulfuric acid method for the determination of carbohydrates in the presence of persulfate. Carbohydr. Polym..

[33] Aramsangtienchai P., Sirirak T., Seesan P., Singrakphon W., Padungkasem J., Srisook K., Watanachote J. (2025). Sulfated polysaccharide from Phyllophorella kohkutiensis: Extraction, purification, and bioactivity assessment. Food Chem. X.

[34] Liu D., Tang W., Yin J.Y., Nie S.P., Xie M.Y. (2021). Monosaccharide composition analysis of polysaccharides from natural sources: Hydrolysis condition and detection method development. Food Hydrocoll..

[35] Beratto-Ramos A., Agurto-Muñoz C., Vargas-Montalba J.P., Castillo R.D.P. (2020). Fourier-transform infrared imaging and multivariate analysis for direct identification of principal polysaccharides in brown seaweeds. Carbohydr. Polym..

[36] Wu H., Zhao Y., Guo Y., Xu L., Zhao B. (2012). Significant longevity-extending effects of a tetrapeptide from maize on *Caenorhabditis elegans* under stress. Food Chem..

[37] Mulio A.T., Chiu C.-S., Chan Y.-J., Lu W.-C., Li P.-H. (2025). New perspectives on polysaccharides from *Gracilaria* and *Gracilariopsis* (Rhodophyta) and their potential applications. Food Front..

[38] Vuai S.A.H. (2022). Characterization of agar extracted from *Gracilaria* species collected along Tanzanian coast. Heliyon.

[39] Guo D., Yu K., Sun X.-Y., Ouyang J.-M. (2018). Structural characterization and repair mechanism of *Gracilaria lemaneiformis* sulfated polysaccharides of different molecular weights on damaged renal epithelial cells. Oxidative Med. Cell. Longev..

[40] Zhang F., Zhang X., Liang X., Wu K., Cao Y., Ma T., Guo S., Chen P., Yu S., Ruan Q. (2022). Defensing against oxidative stress in *Caenorhabditis elegans* of a polysaccharide LFP-05S from Lycii fructus. Carbohydr. Polym..

[41] Sun J., Zhong X., Sun D., Xu L., Shi L., Sui J., Liu Y. (2023). Anti-aging effects of polysaccharides from ginseng extract residues in *Caenorhabditis elegans*. Int. J. Biol. Macromol..

[42] Han B., Sivaramakrishnan P., Lin C.C.J., Neve I.A.A., He J., Tay L.W.R., Sowa J.N., Sizovs A., Du G., Wang J. (2017). Microbial genetic composition tunes host longevity. Cell.

[43] Kumar A., Saha M.K., Kumar V., Bhattacharya A., Barge S., Mukherjee A.K., Kalita M.C., Khan M.R. (2024). Heat-killed probiotic *Levilactobacillus brevis* MKAK9 and its exopolysaccharide promote longevity by modulating aging hallmarks and enhancing immune responses in *Caenorhabditis elegans*. Immun. Ageing.

[44] Jakovljević S., Radojević D., Soković Bajić S., Brdarić E., Mitrović H., Đokić J., Baumann L., Trifunovic A., Golić N., Dinić M. (2026). Bacterial exopolysaccharides mediate activation of flavin-containing monooxygenase 2 to extend lifespan in *Caenorhabditis elegans*. Cell Commun. Signal..

[45] Shi L., Liu Y.-L., Dai M.-N., Ma Y.-X., Wu J.-N., Guo L., Luo L., Fu H.-H., Huang C.-Y., Zhang J.-Y. (2026). D-pinitol extends the lifespan of *Caenorhabditis elegans* through integrated antioxidant defense, proteostasis, and autophagy signaling. npj Aging.

[46] Zioga E., Holdt S.L., Grondahl F., Bang-Berthelsen C.H. (2025). Screening approaches and potential of isolated lactic acid bacteria for improving fermentation of Saccharina latissima. BMC Biotechnol..

[47] Suissa R., Oved R., Maan H., Hadad U., Gilhar O., Meijer M.M., Koren O., Kolodkin Gal I. (2022). Context dependent differences in the functional responses of Lactobacillus strains to fermentable sugars. Front. Microbiol..

[48] Li X., Zhai Z., Hao Y., Zhang M., Hou C., He J., Shi S., Zhao Z., Sang Y., Ren F. (2022). The plasmid encoded lactose operon plays a vital role in the acid production rate of *Lactaceaebacillus casei* during milk beverage fermentation. Front. Microbiol..

